# Traditional Medicinal Plant Extracts and Natural Products with Activity against Oral Bacteria: Potential Application in the Prevention and Treatment of Oral Diseases

**DOI:** 10.1093/ecam/nep067

**Published:** 2011-01-12

**Authors:** Enzo A. Palombo

**Affiliations:** Environment and Biotechnology Centre, Faculty of Life and Social Sciences, Swinburne University of Technology, Hawthorn Victoria 3122, Australia

## Abstract

Oral diseases are major health problems with dental caries and periodontal diseases among the most important preventable global infectious diseases. Oral health influences the general quality of life and poor oral health is linked to chronic conditions and systemic diseases. The association between oral diseases and the oral microbiota is well established. Of the more than 750 species of bacteria that inhabit the oral cavity, a number are implicated in oral diseases. The development of dental caries involves acidogenic and aciduric Gram-positive bacteria (mutans streptococci, lactobacilli and actinomycetes). Periodontal diseases have been linked to anaerobic Gram-negative bacteria (*Porphyromonas gingivalis*, *Actinobacillus*, *Prevotella* and *Fusobacterium*). Given the incidence of oral disease, increased resistance by bacteria to antibiotics, adverse affects of some antibacterial agents currently used in dentistry and financial considerations in developing countries, there is a need for alternative prevention and treatment options that are safe, effective and economical. While several agents are commercially available, these chemicals can alter oral microbiota and have undesirable side-effects such as vomiting, diarrhea and tooth staining. Hence, the search for alternative products continues and natural phytochemicals isolated from plants used as traditional medicines are considered as good alternatives. In this review, plant extracts or phytochemicals that inhibit the growth of oral pathogens, reduce the development of biofilms and dental plaque, influence the adhesion of bacteria to surfaces and reduce the symptoms of oral diseases will be discussed further. Clinical studies that have investigated the safety and efficacy of such plant-derived medicines will also be described.

## 1. Introduction

Oral diseases continue to be a major health problem worldwide [1]. Dental caries and periodontal diseases are among the most important global oral health problems, although conditions such as oral and pharyngeal cancers and oral tissue lesions are also significant health concerns [2]. Despite general advances in the overall health status of the people living in industrialized countries, including oral and dental health, the prevalence of dental caries in school aged children is up to 90% and the majority of adults are also affected [1]. Oral health is integral to general well-being and relates to the quality of life that extends beyond the functions of the craniofacial complex. There is a considerable evidence linking poor oral health to chronic conditions, for example, there is a strong association between severe periodontal diseases and diabetes [1–3]. There is also evidence linking poor oral health and systemic diseases, such as cardiovascular diseases, rheumatoid arthritis and osteoporosis [4], while periodontal diseases and may also contribute to the risk of pregnancy complications, such as preterm low-birth weight [5]. Tooth loss, caused by poor periodontal health (which affects up to 20% of the adult population worldwide) can lead to significant morbidity and premature death [2, 3]. The economic impact of oral diseases is an important consideration with up to 10% of public health expenditure in developed countries related to curative dental care [3]. In most developing countries, expenditure in oral health care is low; access to dental healthcare is limited and is generally restricted to emergency dental care or pain relief [1]. While there has been a marked improvement in oral health in most developed countries worldwide, populations of dentally disadvantaged individuals exist in these countries, often indigenous child populations and those people of low socio-economic status, where oral health is deteriorating [6].

The link between oral diseases and the activities of microbial species that form part of the microbiota of the oral cavity is well established [7]. Over 750 species of bacteria inhabit the oral cavity (∼50% of which are yet to be identified) and a number of these are implicated in oral diseases [7]. The development of dental caries involves acidogenic and aciduric Gram-positive bacteria, primarily the mutans streptococci (*Streptococcus mutans* and *S. sobrinus*), lactobacilli and actinomycetes, which metabolize sucrose to organic acids (mainly lactic acid) that dissolve the calcium phosphate in teeth, causing decalcification and eventual decay. Dental caries is thus a supragingival condition [8]. In contrast, periodontal diseases are subgingival conditions that have been linked to anaerobic Gram-negative bacteria such as *Porphyromonas gingivalis*, *Actinobacillus* sp., *Prevotella* sp. and *Fusobacterium* sp. [7, 9]. In periodontal diseases, the areas at or below the gingival crevice become infected causing a cellular inflammatory response of the gingiva and surrounding connective tissue. These inflammatory responses can manifest as gingivitis (extremely common and seen as bleeding of the gingival or gum tissues) or periodontitis (the inflammatory response results in loss of collagen attachment of the tooth to the bone and in loss of bone) [8].

The global need for alternative prevention and treatment options and products for oral diseases that are safe, effective and economical comes from the rise in disease incidence (particularly in developing countries), increased resistance by pathogenic bacteria to currently used antibiotics and chemotherapeutics, opportunistic infections in immunocompromized individuals and financial considerations in developing countries [9, 10]. Despite several agents being commercially available, these chemicals can alter oral microbiota and have undesirable side-effects such as vomiting, diarrhea and tooth staining [11, 12]. For example, bacterial resistance to most (if not all) of the antibiotics commonly used to treat oral infections (penicillins and cephalosporins, erythromycin, tetracycline and derivatives and metronidazole) has been documented [13]. Other antibacterial agents used in the prevention and treatment of oral diseases, including cetylpyridinium chloride, chlorhexidine, amine fluorides or products containing such agents, are reported to exhibit toxicity, cause staining of teeth or in the case of ethanol (commonly found in mouthwashes) have been linked to oral cancer [14–18]. Hence, the search for alternative products continues and natural phytochemicals isolated from plants used in traditional medicine are considered as good alternatives to synthetic chemicals [19].

## 2. Traditional Plant-Based Medicines

Medicinal plants have been used as traditional treatments for numerous human diseases for thousands of years and in many parts of the world. In rural areas of the developing countries, they continue to be used as the primary source of medicine [20]. About 80% of the people in developing countries use traditional medicines for their health care [21]. The natural products derived from medicinal plants have proven to be an abundant source of biologically active compounds, many of which have been the basis for the development of new lead chemicals for pharmaceuticals. With respect to diseases caused by microorganisms, the increasing resistance in many common pathogens to currently used therapeutic agents, such as antibiotics and antiviral agents, has led to renewed interest in the discovery of novel anti-infective compounds. As there are approximately 500 000 plant species occurring worldwide, of which only 1% has been phytochemically investigated, there is great potential for discovering novel bioactive compounds.

There have been numerous reports of the use of traditional plants and natural products for the treatment of oral diseases. Many plant-derived medicines used in traditional medicinal systems have been recorded in pharmacopeias as agents used to treat infections and a number of these have been recently investigated for their efficacy against oral microbial pathogens. The general antimicrobial activities of medicinal plants and plant products, such as essential oils, have been reviewed previously [22, 23]. Therefore, the purpose of this review is to present some recent examples from the literature of studies that have served to validate the traditional use of medicinal plants with specific biological activity. In particular, traditional medicinal plant extracts or phytochemicals that have been shown to inhibit the growth of oral pathogens, reduce the development of dental plaque, influence the adhesion of bacteria to surfaces and reduce the symptoms of oral diseases will be discussed subsequently. In addition, clinical studies that have investigated the safety and efficacy of such plant-derived medicines will be described.

## 3. Identifying and Evaluating Medicinal Plant Products Used to Treat or Prevent Oral Diseases of Microbial Etiology

**Figure 1:**
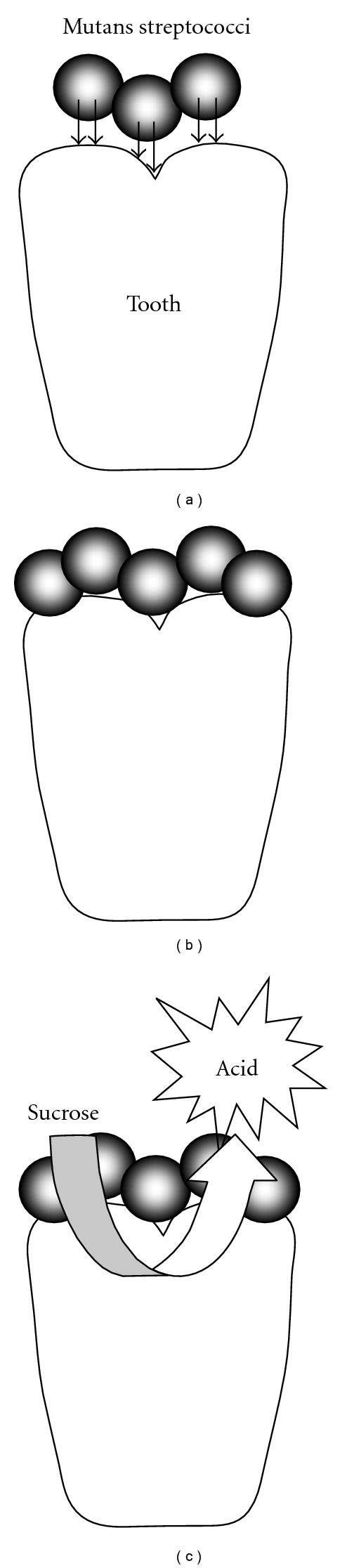
The development of tooth decay. (a) Initial adhesin-mediated attachment of mutans streptococci; (b) aggregation mediated by synthesis of extracellular polysaccharide; (c) metabolism of carbohydrates results in acid production, leading to demineralization and cavitation of the tooth. Plant extracts and phytochemicals have been demonstrated to inhibit any or all of these stages. That is, cidal activity against cariogenic bacteria, inhibition of adherence/aggregation/biofilm formation and inhibition of glycolytic acid production.

Of all oral diseases, the incidence of those that have a microbial etiology is greatest in all parts of the world. Numerous traditional medicinal plants have been evaluated for their potential application in the prevention or treatment of oral diseases. A number of studies have investigated the activity of plant extracts and products against specific oral pathogens, while others have focused on the ability of the products to inhibit the formation of dental biofilms by reducing the adhesion of microbial pathogens to the tooth surface, which is a primary event in the formation of dental plaque and the progression to tooth decay and periodontal diseases [24].  Figure 1 illustrates the stages involved in the development of tooth decay showing the various steps that can be targeted by antimicrobial plant extracts and phytochemicals.

## 4. Antimicrobial Assays

Assays that have been used to test the antimicrobial properties of medicinal plant extracts and natural products used to treat or prevent oral diseases involve assessing the capacity of the test material to inhibit the growth of typical oral pathogens, such as *Streptococcus* sp. (as an example of bacteria implicated in dental caries) and *P. gingivalis* (as an example of bacteria implicated in periodontal diseases). These tests are usually rapid and can be carried out with minimal cost. Agar diffusion methods (disc diffusion or well diffusion) are popular and have been used in a number of studies [19, 25–30]. These methods involve inoculation of the surface of an agar plate with the test micro-organism or pouring molten agar inoculated with the test organism into a Petri dish. The compound to be evaluated can be applied on a paper disc or into a well made in the agar. After appropriate incubation (e.g., in an atmosphere of elevated CO_2_ for streptococci), the appearance of zones of growth inhibition around the disc or well indicates antimicrobial activity. Other tests can involve simple “spot testing” of extracts by placing small quantities (10 or 25 *μ*L) on the surface of agar plates containing an overlay of bacteria and observing inhibition of growth after incubation [9]. Alternative methods involve adsorbing the test compounds in solution to a solid matrix (e.g., silica gel plates) and then overlaying with agar containing indicator organisms [9].

The viable count method can be used as an alternative to agar plate diffusion methods. Cultures of bacteria are centrifuged, washed and resuspended in an appropriate buffer to give a standard concentration of cells. The test compound is added to the cells, the mixture is incubated and the number of viable cells is determined by the pour plate method [11, 12].

Quantifying the antimicrobial activity involves methods analogous to those used to determine the minimum inhibitory concentration [31] of antimicrobial chemical agents such as antibiotics or disinfectants. This involves exposing the test organism to serially diluted extract/compound (either in liquid or solid media) and determining the minimum concentration that inhibits growth [9, 10]. These methods are described in ISO 20776 (Clinical laboratory testing and *in vitro* diagnostic test systems), which states that “Dilution procedures are used to determine the minimum inhibitory concentrations (MICs) of antimicrobial agents and are the reference method for antimicrobial susceptibility testing. In dilution tests, micro-organisms are tested for their ability to produce visible growth on a series of agar plates (agar dilution) or in broth (broth dilution) containing serial dilutions of the antimicrobial agent. The lowest concentration of an antimicrobial agent that, under defined *in vitro* conditions, prevents the appearance of visible growth of a microorganism within a defined period of time is known as the MIC.”

Time-kill assays are used to determine the time required for a compound or extract under investigation to eliminate the growth of the test bacteria [28]. Bacteria are incubated in an appropriate medium in the presence of test material at the minimum bactericidal concentration (MBC). At timed intervals, samples are removed, serially diluted and the number of surviving bacteria is determined by plating on agar media. These data are then used to plot time-kill curves.

Bioautography has been used to carry out preliminary determinations of the number of active constituents in plant extracts. This method involves separation of the extract using thin layer chromatography (TLC) and overlaying the TLC plate with agar inoculated with test bacteria. After appropriate incubation, the presence of the zones of inhibition identifies fractions that contain antimicrobial components [9, 19]. The use of an indicator that is metabolized into a colored product by growing bacteria can help to visualize the zones of inhibition. For example, using tetrazolium red results in the zones of inhibition that appear white to yellow against a red background [9].

## 5. Plant Extracts and Phytochemicals with Activity against Oral Bacteria

There have been numerous *in vitro* studies that have investigated the activity of natural plant substances against oral bacteria. These studies have focused on bacteria known to be involved in the etiology of oral and dental diseases. Early studies have clearly established that a number of substances had potential to be utilized in the dental industry, given their activity against cariogenic bacteria and those bacteria associated with periodontal diseases [32]. Substances that exhibited activity included spice and herb extracts, such as cinnamon bark oil, papua-mace extracts and clove bud oil and constituents of these extracts, such as cinnamic aldehyde and eugenol [32].

## 6. Antibacterial Activity of Crude or Total Plant Extracts

Many studies investigating the activity of traditional medicinal plants against oral pathogens have been limited to examination of crude aqueous or organic solvent extracts. In most cases, the investigators have simply sought to validate the traditional medicinal use of the plant. For example, the use of *Drosera peltata* (Droseraceae) leaves as a traditional treatment for dental caries was validated by a study which showed that chloroform extracts of the aerial plant parts showed broad spectrum activity against numerous bacteria of the oral cavity, with greatest activity against *S. mutans* and *S. sobrinus* (MIC = 31.25 and 15.625 *μ*g mL^−1^, resp.) [25]. Plumbagin was identified as the active component of this extract. Tichy and Novak [9] investigated a collection of 27 medicinal and random plants extracts and identified a number that inhibited the growth of oral streptococci. The most active extracts included those from *Abies canadensis* (Pinaceae), *Albizia julibrissin* (Fabaceae), *Chelidonium majus* (Papaveraceae), *Ginkgo biloba* (Ginkgoaceae), *Juniperus virginiana* (Cupressaceae), *Pinus virginiana* (Pinaceae), *Rosmarinus officinalis* (Lamiaceae), *Sassafras albidum* (Lauraceae), *Tanacetum vulgare* (Asteraceae) and *Thuja plicata* (Cupressaceae). Bioautography indicated that a number of extracts contained common antimicrobial components, while other extracts possessed chemically different constituents.

A boiling water extract of *Coptidis rhizoma* (Ranunculacea), a traditional Chinese medicinal plant, showed bactericidal activity against oral bacteria with particularly good activity against periodontopathogenic bacteria (MIC = 31–250 *μ*g mL^−1^) [33]. Iauk et al. [34] assessed the ability of 10% decoctions and methanol extracts of a number of medicinal plants to inhibit bacterial isolates obtained from crevicular fluid of the periodontal pockets of periodontic patients. In general, the methanol extracts showed greater activity than the decoctions. The extract from the leaves of *Hamamelis virginiana* (Hamamelidaceae) had the greatest overall activity against all bacteria tested, with MIC values generally <512 *μ*g mL^−1^, particularly against *Porphyromonas* spp., *Preveotella* spp. and *Actinomyces odontolitycus*.

Garlic, *Allium sativum* (Liliaceae), has been used as a medicine since ancient times because of its antimicrobial properties [28]. While garlic has been shown to have activity against a wide range of bacteria, the specific activity against the Gram-negative oral pathogens, including *P. gingivalis* has only recently been demonstrated [28]. A garlic extract containing the major antimicrobial component, allicin, was active toward Gram-negative pathogens (MIC = 1.1–17.4 mg mL^−1^) but less active against Gram-positive bacteria (MIC = 35.7–142.7 mg mL^−1^). The extract almost completely inhibited trypsin-like protease activity (implicated in the pathogenesis of periodontitis) of *P. gingivalis*. Taken together, these observations suggest that garlic extract or allicin may be of therapeutic use against periodontal diseases or other oral diseases [28].

*Harungana madagascariensis* (Hypericaceae) is a native African plant with antimicrobial properties that contains numerous antimicrobial components [35]. Using successive Soxhlet solvent extractions, an ethyl acetate extract of leaves was prepared and tested against numerous oral pathogens. While the extract was able to kill all oral bacteria tested (including *Actinomyces*, *Fusobacterium*, *Lactobacillus*, *Prevotella*, *Propionibacterium* and *Streptococcus* species), poly (d,l-lactide-co-glycolide) nanoparticles containing extract showed enhanced activity. The authors suggested that this may have been due to the bioadhesive properties of the polymer resulting in the extract being in contact with the bacteria for prolonged periods [35].

The resin exuded by the *Pistacia lentiscus* (Anacardiaceae) tree, known as mastic gum, is used in the preparation of foods and as a remedy for oral malodor and has been shown to have antimicrobial activity. The activity of a mastic gum extract against *P. gingivalis* was demonstrated using disc diffusion assays, but its low solubility suggested that it may be useful for local application rather than as a mouthrinse [36]. The antibacterial activity of *Pistacia vera* extracts against oral streptococci has also been demonstrated (MIC > 1 mg mL^−1^), including the inhibition of adherence and glycolysis [37].

Crude ethanol extracts of *Piper cubeba* (Piperaceae) exhibited good antimicrobial properties against a range of cariogenic pathogens (MIC = 90–200 *μ*g mL^−1^), although no information about the activity against periodontal pathogens was provided [38].

Cold and hot water and ethanolic extracts of *Breynia nivosus* (Euphorbiaceae) and *Ageratum conyzoides* (Asteraceae) were tested for activity against *S. mutans* [30]. While the hot water and ethanol extracts (at >25 mg mL^−1^) of *B. nivosus* showed activity, none of the *A. conyzoides* extracts were active. However, combining the ethanolic extracts of both plants resulted in synergistic activity. Administration of 250–5000 mg kg^−1^ of *B. nivosus* extract to mice did not lead to mortality or produce acute toxicity after 24 h.

Smullen et al. [39] determined the ability of commercially available extracts or fresh aqueous propanone extracts (PE) of foods with high polyphenol content to inhibit the growth of *S. mutans* and other oral pathogens. All the extracts showed activity, with the PE of red grape seeds exhibiting the greatest activity against *S. mutans* (MIC = 500 *μ*g mL^−1^). The green tea and unfermented cocoa PE were most active against other oral pathogens. Overall, the commercial extracts were not as active as the PE. Various PE were also able to prevent adhesion of *S. mutans* to glass. These data suggest that extracts of polyphenol-containing foods may have a preventative role against dental caries.

*Helichrysum italicum* (Compositae) is widely found in the Mediterranean region and has been shown to have a variety of biological properties. An ethanol extract of powdered flowering tops was found to exert antimicrobial activity against *S. mutans*, *S. sanguis* and *S. sobrinus*, with MIC values of 31.25–62.5 *μ*g mL^−1^ [40].

Two recent studies have examined a number of plants traditionally used in Brazil [41] or South Africa [42], respectively, for activity against oral pathogens. All four Brazilian plant extracts, *Cocos nucifera* (Palmae), *Ziziphus joazeiro* (Rhamnaceae), *Caesalpinia pyramidalis* (Fabaceae) and *Aristolochia cymbifera* (Aristolochiaceae), were active against the test bacteria, with the ethanol extract of *A. cymbifera* being the most effective (MIC = 0.1–4.0 mg mL^−1^). Of the South African plants tested, all were effective (MIC > 25 mg mL^−1^), although the high therapeutic indices determined for extracts of *Annona senegalensis* (Ammonaceae), *Euclea natalensis* (Ebenaceae) suggested that these may be safe to administer *in vivo*.

## 7. Antibacterial Activity of Propolis

Propolis has been shown to exhibit good antimicrobial activity against a range of oral bacteria and inhibit the adherence of *S. mutans* and *S. sobrinus* to glass [26]. It was also shown to be a potent inhibitor of water-soluble glucan synthesis. In the same study, an extract of *Arnica montana* (Asteraceae) demonstrated no antimicrobial activity, nor did the extract affect adherence or glucan synthesis. Uzel et al. [43] also investigated the activity of propolis against a number of microorganisms, including *S. mutans* and *S. sobrinus*. Ethanol extracts of four samples of propolis collected from different geographical regions in Anatolia exhibited MIC values of 2–64 *μ*g mL^−1^, with activity likely to be due to the presence of numerous flavonoids. Propolis showed antimicrobial activity similar to chlorhexidine and greater than clove or sage extracts in a study investigating the ability of these chemicals to inhibit the growth of microbes obtained from the saliva of periodontally healthy subjects and those with chronic perodontitis [44]. *Nidus Vespae*, the honeycomb of *Polistes olivaceous* (De Geer), *P. japonicus* de Saussure and *Parapolybiavaria fabricius*, is a traditional Chinese medicine that has a number of pharmacological properties. While *Nidus Vespae* is similar to propolis, it contains additional material including waxes and aromatic oils. Like propolis, extracts and fractions of *Nidus Vespae* have been shown to exert antimicrobial activity toward a number of oral microorganisms, in particular *S. mutans* [45]. In addition, the extracts showed significant anti-acidogenic activity.

## 8. Antibacterial Activity of Purified Phytochemicals

The following section describes the studies of phytochemicals that have been shown to be active against oral pathogens. The studies are grouped according to the general class of phytochemicals investigated.

### 8.1. Flavonoids and Other Polyphenols

In a study of a number of methanolic plant extracts, two active isoprenylflavones, artocarpin and artocarpesin, were isolated from *Artocarpus heterophyllus* (Moraceae). These inhibited the growth of numerous cariogenic and oral bacteria, including mutans and other oral streptococci, actinomyces and lactobacilli, at MIC values of 3.13–12.5 *μ*g mL^−1^ [46]. Flavonone phytoalexins from *Sophora exigua* (Leguminosae) have been shown to inhibit the growth of numerous cariogenic bacteria, with 5,7,2′,4′-tetrahydroxy-8-lavandulylflavanone being the most active [47]. *Erythrina variegata* (Leguminosae) is used in folk medicine in tropical and subtropical regions and displays a number of biological properties, including antibacterial activity [48]. Seven isoflavonoids isolated from the roots of this plant were tested for their ability to inhibit the growth of cariogenic oral bacteria. Of these, erycristagallin showed the most potent inhibitory activity with MIC values of 1.56–6.25 *μ*g mL^−1^. In addition, erycristagallin completely suppressed the incorporation of radio-labeled thymidine and glucose in *S. mutans*, suggesting that the compound interferes with bacterial uptake of metabolites [48].

The root bark of *Morus alba* (Moraceae) has been used as a traditional medicine in Asian countries and exhibits antibacterial activity against food poisoning micro-organisms [11]. Using activity against *S. mutans* in bioassay-guided fractionation of a methanol extract of dried root bark, and organic solvent fractions of this extract, the active antibacterial constituent was identified as kuwanon G. The compound displayed an MIC of 8 *μ*g mL^−1^ against *S. mutans*, which was comparable to chlorhexidine and vancomycin (1 *μ*g mL^−1^). Time-kill assays indicated that *S. mutans* was completely inactivated by 20 *μ*g mL^−1^ kuwanon G within 1 min, while testing against other bacteria suggested that the compound displayed preferential antimicrobial activity against cariogenic bacteria. Electron microscopic examination of *S. mutans* cells treated with kuwanon G indicated that the mode of antibacterial action was inhibition or blocking of cell growth, as treated cells showed a disintegrated surface and an unclear cell margin. A similar mode of antibacterial action has been reported for the compound isopanduratin A isolated from *Kaempferia pandurate* (Zingiberaceae) [49].

A number of components of tea, *Camelia sinensis* (Theaceae), exhibit anticariogenic effects through various modes of action, including bactericidal effects on oral bacteria, prevention of adherence of bacteria to tooth surfaces, inhibition of glucan production and inhibition of amylases (reviewed in [50]). Monomeric polyphenols, in particular simple catechins such as epicatechin, epicatechin gallate, and epigallocatechin gallate are believed to be responsible for these biological effects [50, 51].

The paste of tender leaves of *Psidium guajava* (Myrtaceae) has been used traditionally to maintain oral hygiene, while other parts of the plant have various bioactive properties [19]. A methanol extract of *P. guajava* leaves was shown to exhibit inhibitory activity against two strains of *S. mutans*. Fractionation guided by bioautography yielded the active compound, quercetin-3-*O*-*α*-l-arabinopyranoside or guaijaverin, which had MIC values of 2–4 mg mL^−1^. At sub-MIC values, guaijaverin was also able to inhibit acid production of the test bacteria, decrease the hydrophobicity of one of the bacteria and inhibit the adherence of both bacteria to glass. The anti-adherent properties of this plant were supported by the reduction of cell-surface hydrophobicity observed in “early settler” plaque bacteria (*S. mitis*, *S. sanguinis* and *Actinomyces*) exposed to 1 mg mL^−1^ *P. guajava* extract [52]. Recently, a proteomics approach was used to show that treatment of *S. mutans* with a low concentration (1.6%, v/v) of a *Psidium cattleianum* water extract resulted in the downregulation of genes involved in lactic acid production, general metabolism and glycolysis [53]. At higher concentrations (25–100%, v/v), the extract was able to inhibit *S. mutans* biofilms.

Malvidin-3,5-diglucoside (malvin) was identified as the active constituent of an ethanol extract of *Alcea longipedicellata* (Malvaceae) responsible for activity against oral streptococci, with MIC values of 160–200 *μ*g mL^−1^ [54]. The compound macelignan was isolated from *Myristica fragrans* (Myristicaceae) and shown to exert antimicrobial activity against *S. mutans* (MIC = 3.9 *μ*g mL^−1^, MBC = 7.8 *μ*g mL^−1^) comparable to chlorhexidine and superior to other natural anticariogenic agents (sanguinarine, eucalyptol, thymol, menthol and methyl salicylate) [12]. Macelignan (20 *μ*g mL^−1^) displayed rapid antibacterial activity and completely eliminated viable *S. mutans* within 1 min. It also showed preferential activity against other cariogenic bacteria. Macelignan also displayed antibiofilm activity against *S. mutans*, *S. sanguis* and *A. viscosus* [55].

The antibacterial activities of an ethanol extract of the seeds of *Piper cubeba* were described above. A plant compound, (−)-cubebin, a naturally found derivative and three semi-synthetic derivatives were evaluated against a number of Gram-positive oral bacteria and the yeast *Candida albicans* [38]. While bacteriostatic and/or fungicidal activity was observed with all test compounds (although at levels well above those of chlorhexidine), no information concerning the activity against Gram-negative pathogens was provided.

Naringin, a polymethoxylated flavonoid commonly found in citrus fruit and an FDA-approved health supplement, was shown to inhibit the growth of periodontal pathogens and other common oral microorganisms (9.8–125 mg mL^−1^) [56]. Using time-kill assays, naringin was shown to be particularly effective against *Actinobacillus actinomycetemcomitans* and *P. gingivalis* with significant growth inhibition within 3 h and greater inhibition with increasing incubation time and naringin concentration.

### 8.2. Terpenes

Bakuchiol isolated from the Chinese medicinal plant, *Psoralea corylifolia* (Fabaceae), has shown activity against numerous Gram-positive and Gram-negative oral pathogens (MIC = 1–4 *μ*g mL^−1^). It was able to inhibit the growth of *S. mutans* under a range of sucrose concentrations, pH values and in the presence of organic acids in a temperature-dependent manner and also inhibited the growth of cells adhered to a glass surface [57].

Liu et al. [58] purified seven new *ent*-rosane diterpenoids and a new labdane diterpene from the Chinese medicinal plant, *Sagittaria sagittifolia* (Alismaceae). Four of these compounds (sagittine A–D) exhibited antibacterial activity against *S. mutans* and *Actinomyces naeslundii* (with MIC values of between 62.5 and 125 *μ*g mL^−1^) while another (Sagittine E) was only active against *A. naeslundii* (MIC = 62.5 *μ*g mL^−1^). Recently, the same group identified five new diterpenoids from *Sagittaria pygmaea* [59]. None of these displayed activity against *A. actinomycetemcomitans*, while four of the others were active against *A. viscosus* and three were active against *S. mutans*, of Which 18-*β*-d-3′,6′-diacetoxyglucopyranosyl-*ent*-kaur-16-ene was the most active (MIC = 15.6 *μ*g mL^−1^).

*Curcuma xanthorrhiza* (Zingiberaceae) has traditionally been used to treat a number of disorders. Xanthorrhizol, isolated from a methanol extract of the plant roots, was shown to have high levels of antibacterial activity against oral pathogens, in some cases equal or similar to that of chlorhexidine, for example MIC against *Streptococcus* sp. is equal to 2–4 *μ*g mL^−1^ [60].

### 8.3. Alkaloids

The alkaloid berberine isolated from *C. rhizoma* (Ranunculacea) showed bactericidal activity against oral bacteria, with greatest activity against *A. actinomycetemcomitans* (MIC = 13 *μ*g mL^−1^) and *P. gingivalis* (MIC = 20 *μ*g mL^−1^), although much less activity was observed against *Lactobacillus* and *Streptococcus* species. Berberine also inhibited the collagenase activity of *A. actinomycetemcomitans* and *P. gingivalis* [33].

### 8.4. Sugar Alcohols

Xylitol is a sugar alcohol naturally found in plants that is used as an artificial sweetener in many foods [61]. Its anticariogenic properties were investigated by adding 0.78–50% xylitol to broth cultures of *S. mutans*, *S. saliviarius* and *S. sanguis*, incubating at 37°C for 18 h and determining the optical density of the cultures. *Streptococcus mutans* was the only bacterium significantly inhibited by xylitol at 1.56%, while all bacteria showed statistically significant inhibition at levels above 1.56%. The study concluded that xylitol exhibited anticariogenic effects by inhibiting the growth of *S. mutans* while not affecting other streptococci that are part of the normal oral flora [61].

### 8.5. Other Phytochemicals

Several constituents found in hops, *Humulus lupulus* (Cannabaceae), have been found to display antibacterial activity against *S. mutans*, *S. saliviarius* and *S. sanguis* in disc diffusion assays [62]. The MICs of beta acid, xanthohumol (a flavonoid), tetra iso-*α* acid, iso-*α* acid and thymol (a terpene) against *S. mutans* were determined as 2.0, 12.5, 12.5, 50 and 150 *μ*g mL^−1^, respectively. These antibacterial activities were enhanced in the presence of ascorbic acid or when pH was lowered, suggesting that this effect was the result of the acidic nature of ascorbic acid. Furthermore, the antibacterial activity of 20% ethanol was found to increase in the presence of beta acid or thymol.

The antimicrobial properties of a number of commercially available dentifrices containing herbal products have been evaluated against oral pathogens [63]. The antimicrobial effectiveness of the products varied greatly; 10 of the 14 products showed activity against all test bacteria (*S. mutans*, *S. sanguis* and *A. viscosus*), but only 6 of these were able to inhibit the yeast *C. albicans*. The observations that some of the dentifrices exhibited no antimicrobial activity and that some appear to have been contaminated by other microorganisms were of particular concern.

Thirty-nine natural products were tested for their antibacterial properties against four strains of *S. mutans* and their ability to prevent adherence of the bacteria [10]. Catechol was the most active and inhibited both the growth (MIC = 6.5 *μ*g mL^−1^) and adherence (>75% inhibition) of all test bacteria, while emetine hydrochloride and quinidine sulfate inhibited three of the bacterial strains, but not always to the same extent as catechol.

## 9. Investigating Bacterial Adhesion to Surfaces: Reducing Biofilm and Plaque Formation

Dental plaque is an example of a specialized bacterial biofilm that develops on the surface of teeth, dental restorations, prostheses and implants [64]. The colonizing bacteria that make up the biofilm consortium produce organic acids, such as lactic acid, by metabolizing carbohydrates that results in the formation of dental caries [11]. Plaque is also involved in the etiology of periodontal diseases [65]. A number of methods have been used to evaluate the potential of natural products to reduce or eliminate oral biofilms. In contrast to the methods used to investigate antimicrobial properties (which involve killing of test bacteria), these methods are used to assess the ability of the test products to prevent the adsorption or adhesion of bacteria to surfaces (the initial stage of biofilm development) or to prevent the synthesis of the insoluble layer of polysaccharides (fructans and glucans) by sucrose-dependent fructosyltransferase and glucosyltransferase activity of the colonizing bacteria (the second and irreversible stage of biofilm development) [12, 24]. These enzymes split sucrose and transfer the glucose moiety to a glucose polymer, forming strongly adhesive extracellular polysaccharides that allow the bacteria to attach to tooth surfaces [8]. Anti-adhesion agents may be preferable to those that are bactericidal as selective pressure leading to resistance and overgrowth of resistant bacteria would be avoided [24].

Bacterial adhesion can be evaluated by a number of assays. Radioactively labeled bacteria are added to saliva- or protein-coated hydroxyapatite (HA) beads in the presence of the test compound. After incubation, the HA is washed to remove the loosely bound bacteria and samples counted in a scintillation counter [24, 65–67]. The adhesion to protein-coated surfaces can also be investigated using microtiter plates in which the wells are coated with collagen, human fibrinogen or human serum and bacteria together with test material (at different concentrations) are added. After incubation, unbound bacteria are removed by aspiration, the adherent cells fixed with methanol, stained with crystal violet and the absorbance at 550 nm recorded [29, 66]. Adhesion to glass surfaces can be assessed in a similar manner [66, 68].

The inhibition of biofilm formation by oral pathogens has been assessed by cell culture plate assays where bacteria are incubated in the presence or absence of test compounds [29, 65]. After appropriate incubation, free bacteria are removed from microtiter wells by aspiration, biofilms are stained with crystal violet and the absorbance measured to indicate the level of biofilm formation. Scanning electron microscopy has been used to visibly assess the extent of biofilm formation in the presence and absence of test material [29]. Alternatively, the inhibitory effects on biofilms have been assessed by measuring the level of activity of bacterial enzymes synthesized during oral biofilm formation, specifically fructosyltransferase and glucosyltransferase [24, 66], or the activity of glucan-binding lectin [66].

## 10. Plant Extracts and Phytochemicals Which Inhibit the Adhesion of Oral Bacteria

As a part of some of the studies described above, plants extracts and phytochemicals were investigated for their ability to prevent adhesion of cariogenic bacteria to surfaces. In the following section, studies which have investigated this activity as a major objective are discussed, even though antibacterial properties may have also been investigated.

### 10.1. Anti-Adhesion Activity of Crude or Total Plant Extracts

Cranberry, *Vaccinium macrocarpon* (Ericaceae), has been recognized for its beneficial effects on human health, including the prevention urinary tract infections by interfering with adhesion of *Escherichia coli* to cells and preventing adhesion of *Helicobactor pylori* to gastric mucosa [69, 70]. Numerous studies have investigated the ability of cranberry juice or cranberry constituents to prevent adhesion of oral pathogens to surfaces and related phenomena, such as the production of glucans and fructans, and the formation of biofilms. Exposure of oral streptococci to 25% cranberry juice for as little as 10 s has been shown to inhibit adsorption of cells to saliva-coated hydroxyapatite beads by between 61.8% and 95.1%, with the exception of *S. sobrinus* for which reduced adsorption was seen after 10 min [65]. In addition, cell surface hydrophobicity of some of the bacteria was reduced and a preparation of high molecular weight cranberry juice constituents inhibited biofilm formation. These data indicated that cranberry juice could prevent dental plaque development by inhibiting the initial phase of biofilm formation. A preparation of high molecular weight cranberry material was also shown to reduce the activity of fructosyltransferase and glucosyltransferase [24] and promote the desorption of *S. sobrinus* biofilms, especially those formed by nonsucrose-dependent adhesion in the absence of polysaccharides [71]. Bacteria pre-exposed to high molecular weight cranberry material produced thinner biofilms with reduced bacterial density. Similar results were obtained with *P. gingivalis*, in that a high molecular weight cranberry fraction inhibited biofilm formation while not affecting growth and viability of bacteria [29]. At concentrations of 62.5 *μ*g mL^−1^ and higher, the fraction significantly inhibited biofilm formation, yet no effect on bacterial growth or biofilm viability was apparent with concentrations up to 250 *μ*g mL^−1^. However, this treatment did not result in biofilm desorption. The specific inhibitory effect of cranberry juice on *S. mutans* biofilm formation has been suggested to be related to the inhibition of glucan-related processes (inhibition of glucosyltransferase, blocking of bacterial adhesion mediated by surface glucans and reduction of insoluble glucan content) [70]. Recently, the cranberry juice constituents active against *S. mutans* biofilms have been identified as polyphenols, specifically proanthocyanidins and flavonols [72].

*In vitro* experiments showed that cacao bean husk extract markedly reduced the growth rate (69–72% reduction) and inhibited insoluble glucan synthesis of *S. mutans* and sucrose-dependent adhesion of *S. mutans* and *S. sobrinus* to a glass surface (85% inhibition at >5 mg mL^−1^) [73]. In addition, *in vivo* experiments in pathogen-free rats infected with these bacteria indicated that the extract exhibited significant cariostatic activity.

Two methods, adherence to glass and adherence to saliva-coated hydroxyapatite (S-HA), were used to assess the effect of ethanol extracts of six plants on the adherence of *S. mutans* [66]. Extracts of *Andrographis paniculata* (Acanthaceae), *Cassia alata* (Leguminosae), *Camellia sinensis*, *Psidium guava* and *Harrisonia perforata* (Simaroubaceae) were able to inhibit one or both strains of *S. mutans* tested using both methods, although relatively high concentrations were required (MIC_50_ values of 3–5 mg mL^−1^). Overall, the active extracts were less effective in inhibiting adhesion to S-HA than to glass, suggesting that salivary glycoproteins are important in bacteria-surface interactions, leading to stronger adherence to S-HA. *Camellia sinensis* extract exhibited the greatest inhibition of glucosyltransferase activity while *Andrographis paniculata* extract showed greatest inhibition of glucan-binding lectin activity of both strains.

As mentioned above an ethanol extract of *Helichrysum italicum* (Compositae) powdered flowering tops was found to exert antimicrobial activity against *S. mutans*, *S. sanguis* and *S. sobrinus* [40]. At sub-MIC levels (<31.25 *μ*g mL^−1^), the extracts were able to reduce cell surface hydrophobicity, adherence to glass and cellular aggregation of *S. mutans* in the presence of dextran.

Plants of the genus *Mikania* (Asteraceae) are used medicinally in Brazil because of their numerous pharmacological properties. Extracts and fractions of the aerial parts of *Mikania laevigata* and *M. glomerata* have been shown to inhibit the growth of mutans streptococci (MIC = 12.5–100 *μ*g mL^−1^) and sucrose-dependent adherence of cells to a glass surface [68]. Analysis of the hexane fraction of the extracts, which displayed the most potent activities, indicated that the active components have nonpolar characteristics.

Methanol extracts of the roots of *Polygonum cuspidatum* (Polygonaceae), traditionally used in Korea to maintain oral health, were shown to reduce the viability of *S. mutans* and *S. sobrinus* (MIC = 0.5–4 mg mL^−1^), as well as inhibit sucrose-dependent adherence, water-insoluble glucan formation, glycolytic acid production and acid tolerance [74]. The authors suggested that inhibitory effects may be mediated by the presence of alkaloids, phenolics and sterol/terpenes in the extract.

Aqueous and methanol extracts of cloves from *Syzygium aromaticum* (Myrtceae) were shown to affect the cariogenic properties of *S. mutans*, as exhibited by the ability of the extracts to inhibit adhesion of the bacteria to glass, reduce cell surface hydrophobicity and inhibit the production of glucosyltransferase [75]. While some differences in the activities of the aqueous and solvent extracts were observed, overall this study showed that the two extracts were equally effective.

Crude aqueous extracts of *Piper betle*, a plant used traditionally in the control of dental and oral diseases in South East Asia, have been shown to inhibit the growth, adherence and glucan production of *S. mutans* [76]. Further studies showed that acid production was also inhibited, while microscopic examination of bacterial cells exposed to extract showed cells with “nucleoid material coagulated into thick electron-dense filaments and destruction of the plasma cell membrane and inner cell wall” which was more apparent with higher extract concentrations [77]. Bioautography identified one major phenolic constituent as the antimicrobial component of the extract [77].

### 10.2. Anti-Adhesion Activity of Purified Phytochemicals

An investigation of the effects of macrocarpals (phloroglucinol-sesquiterpene-coupled compounds) extracted from eucalyptus leaves on periodontopathic bacteria demonstrated that these compounds were able to inhibit the growth of the majority of bacterial strains tested [67]. *Porphyromonas gingivalis* was the most sensitive bacterium with an MIC of 1 *μ*g mL^−1^ for macrocarpals A and B, and 0.5 *μ*g mL^−1^ for macrocarpal C. In addition, all three compounds were able to inhibit the expression of *P. gingivalis* proteases and the binding of cells to S-HA by 70–80% at a concentration of 10 *μ*g mL^−1^.

As mentioned above, xanthorrhizol has been shown to inhibit the growth of oral pathogens. The ability of xanthorrhizol to inhibit bioflims of *S. mutans* has also been investigated [78]. Using 4-, 12-, 20- and 24-h old bioflims, the greatest level of inhibition was observed at the adherent (4 h) stage (complete inhibition at concentrations of 5, 10 and 50 *μ*mol l^−1^). In older biofilms, 76% inhibition was achieved with 50 *μ*mol l^−1^ and an exposure time of 60 min.

**Figure 2:**
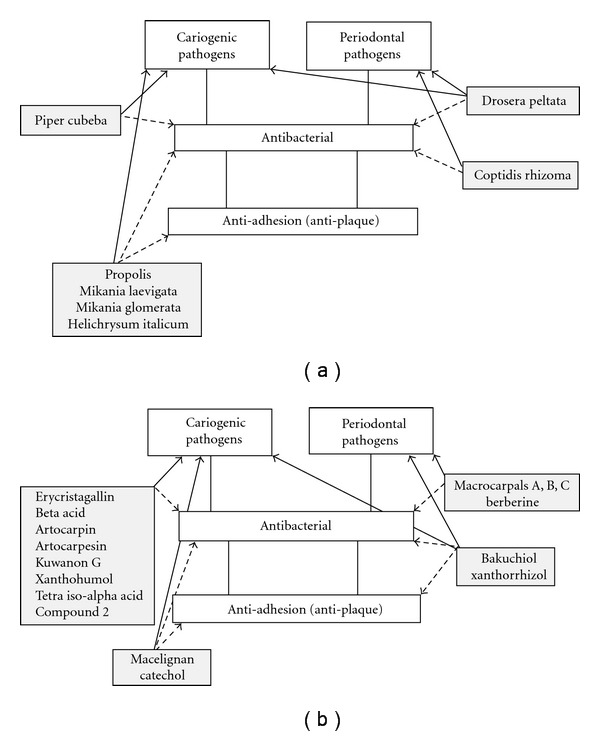
Potential application of plant extracts (a) and phytochemicals (b) in the prevention and treatment of oral diseases caused by cariogenic and periodontal microbial pathogens. Where known, the likely uses of extracts and phytochemicals are indicated with respect to their target pathogens (solid arrows) and biological activities (dashed arrows).

The activities of plant extracts and phytochemicals detailed in the sections above do not necessarily indicate that all of these products will have clinical value. In fact, many of the products are effective only at relatively high concentrations. Indeed, Cos et al. [79] have suggested that strict criteria should be used to assess the potential application of natural products. In the context of anti-infective agents, MIC levels of <100 *μ*g mL^−1^ are indicative of useful bioactivity for natural product extracts. Numerous studies have compared active compounds to currently used antibacterial compounds used in dentistry, such as chlorhexidine and triclosan, as a way of determining relative effectiveness. The MIC of chlorhexidine is *∼*1 *μ*g mL^−1^ [49] while triclosan has an MIC of 0.1–20 *μ*g mL^−1^ [80]. Using the above criteria, extracts with MIC values of ≤100 *μ*g mL^−1^ and isolated phytochemicals with MIC values of ≤20 *μ*g mL^−1^ may be considered useful for the development as products for application against oral infections. These are listed in Table 1 and their specific applications in the prevention or treatment of oral infections (i.e., antibacterial or antiplaque properties) are summarized in Figure 2.

### 10.3. Essential Oils with Activity against Oral Bacteria

The antibacterial properties of essential oils are well-known and activity against bacteria found in the oral cavity, including pathogens, has been documented [23]. Indeed, there is evidence that commercial mouthwashes containing essential oils are useful in the long-term control of plaque and mild-to-moderate gingivitis and are preferred to those containing chlorhexidine for long-term daily use [81, 82]. A number of recent studies add to the evidence that essential oils may be suitable additives in products used for the maintenance of oral hygiene or prevention of dental disease.

The essential oil of *Melaleuca alternifolia* (Myrtaceae), known as tea tree oil (TTO), has been used medicinally for many years. TTO has antimicrobial properties and is used in the superficial treatment of skin infections. The activity of TTO against an extensive collection of oral bacterial isolates was investigated by Hammer et al. [31] who determined MIC and MBC values in the range 0.003–2.0% (v/v). Further, time-kill assays showed that exposure of *S. mutans* and *Lactobacillus rhamnosus* to 0.5% (v/v) TTO resulted in >3 log reduction of viable cells within 30 s. The activity of TTO against oral pathogens was supported in a study involving this and other essential oils, including manuka oil, eucalyptus oil, lavandula oil and rosmarinus oil [83]. In addition to their inhibitory and bactericidal activities, most of the oils were able to inhibit the adhesion of *S. mutans* and *P. gingivalis*.

Essential oils are also capable of enhancing the activity of chlorhexidine. When used in combination, the essential oils of cinnamon and manuka were able to significantly reduce the amount of chlorhexidine required to inhibit the growth of oral pathogens [84]. This enhanced activity was also seen against bacterial cultures grown as biofilms. Between 4- and 10-fold reductions of the amount of chlorhexidine required to inhibit biofilm bacteria was observed when used in combination with cinnamon, manuka and *Leptospermum morrisonii* oils.

The essential oils of *Artemisia lavandulaefolia* (Asteraceae), *A. capillaries*, *A. scoparia* and *A. feddei* have been shown to inhibit the growth of oral bacteria [85–87], with the greatest activity generally observed against obligate anaerobes. However, the oils also showed strong activity against other groups, including facultative anaerobes and microaerophilic bacteria. A recent study reported that the essential oil of *Cryptomeria japonica* (Taxodiaceae) exhibited strong activity against all bacteria tested, especially oral bacteria, with MIC of 0.025–0.5 mg mL^−1^ [87].

While these *in vitro* results are very encouraging, the known toxicity of TTO when ingested [88] suggests that further studies of the safety of this and other essential oils for use in the oral cavity need to be addressed. In this context, Takarada et al. [83] showed that the essential oils used in their study had little effect on human umbilical vein endothelial cells *in vitro* when tested at a concentration of 0.2% (v/v), well within the MIC and MBC values of several oils against some of the bacteria tested.

## 11. *In Vivo* Testing of Dental Products Containing Plant-Derived Chemicals

The majority of the studies described above have involved *in vitro* assessment of plant extracts and chemicals against oral pathogens. While these studies provide important information about the potential use of these products, the studies described below have examined their *in vivo* efficacy in human clinical trails.

In a one group time series design and single blind study, a mouthrinse containing an extract of the leaves of *Streblus asper* (Moraceae) tested in 30 volunteers resulted in a significant (*P* < .05) and selective reduction in salivary *S. mutans* (assessed by determining the viable bacterial counts in saliva samples) with no effect on total salivary bacteria [89]. Moreover, no effect on salivary pH and buffer capacity was observed. Similarly, a randomized placebo-controlled clinical study of a mucoadhesive dental gel containing an extract of *Azadirachta indica* (Meliaceae) involving 36 participants showed a significant reduction (*P* < .05) in salivary *S. mutans* and *Lactobacillus* sp. bacterial counts after 6 weeks (test group) compared to the placebo group, which was greater than the reduction seen with a chlorhexidine mouthwash (positive control group) [90]. A significant reduction in plaque index was also observed (*P* < .05). *In vivo* evaluation of a 2.5% garlic mouthwash using 30 subjects indicated that the solution was able to maintain a reduced level of oral bacteria and mutans streptococci 2 weeks after use, although adverse effects such as unpleasant taste, halitosis and nausea were reported [91]. This 5-week study measured bacterial levels at baseline (Week 1), and the reduction in bacterial numbers after using a vehicle solution as a mouthwash (Week 2), a garlic mouthwash (Week 3) and in the following 2 weeks. A significant difference (*P* < .05) from baseline levels of bacteria was observed only for the period following treatment (the residual effect) and not during the treatment (garlic mouthwash) period.

A randomized placebo-controlled study in which two commercial tea extracts were used as mouthrinses showed that each was able to significantly reduce the microbial load of the oral cavity [92]. A significant reduction (*P* < .05) in the number of bacteria per milliliter of liquid expectorated 5 and 60 min after gargling for 60 sec was observed. Combining the extracts with the surfactant lauryl sodium sulfate resulted in a potentiation of antimicrobial action, probably as a result of the combined effects of the surfactant and tea polyphenols on microbial cell walls and membranes.

A randomized controlled study utilizing a single-blind cross-over design involving 29 participants demonstrated that twice daily rinsing with an essential oil-containing mouthrinse led to significant reductions (*P* < .01) in total recoverable streptococci and total recoverable *S. mutans* (70 and 75%, resp.) in interproximal plaque. Saliva samples also showed significant reductions in the recoverable levels of total streptococci and *S. mutans* (*P* < .001 and *P* = .012, resp.) [93].

A single-blind, randomized cross-over study involving 15 volunteers demonstrated that chewing sticks from *Salvadora persica* had similar effects on the levels of subgingival plaque microbiota as regular toothbrushing without toothpaste [94]. However, the level of *A. actinomycetemcomitans* was significantly reduced (*P* < .05) by the use of chewing sticks. In a similar study investigating a commercial herbal mouthwash containing *Salvadora persica* extract, significant reductions (*P* < .01) in gingival bleeding were observed in both test and placebo subjects. However, a significant reduction (*P* < .05) in the carriage of cariogenic bacteria was observed only in the test subjects [95].

The efficacy of a hydroalcoholic extract (HAE) of pomegranate, *Punica granatum* (Punicaceae), in the prevention of dental plaque was investigated in a randomized controlled study involving 60 participants who were randomized into three groups: HAE, chlorhexidine (positive control) or distilled water (negative control) [96]. Dental plaque material was collected from each participant before and after a 1-min mouth rinse with the specified material and the number of viable bacteria determined by a standard plate count method. A significant reduction in bacteria was observed in the HAE and chlorhexidine groups (*P* < .0001) compared to the control group (distilled water). The activity of HAE was maintained for at least an hour. The authors concluded that pomegranate extract could be useful in the prevention of diseases caused by plaque bacteria. These results are supported by an *in vitro* study of a phytotherapeutic gel containing *P. granatum* plant powder [97], which was able to inhibit the adherence of *S. mutans*, *S. mitis* and *S. sobrinus* (as well as *C. albicans*) to glass in the presence of sucrose.

While these studies demonstrate the potential *in vivo* use of plant extracts in maintaining oral hygiene, a randomized controlled study of two herbal mouthrinses containing extracts of *Juniper communis* (Cupressaceae), *Urtica dioica* (Urticaceae) and *Achillea millefolium* (Asteraceae) using subjects with moderate gingival inflammation found no clinically measurable effect on plaque growth (*P* = .325) and gingival health (*P* = .723), despite the fact that *in vitro* tests showed weak antimicrobial activity against oral pathogens [98]. In addition, a study involving 15 participants in a Phase I trial and 17 participants in a Phase II trial of a novel mouthrinse containing essential oils and extracts from *Melaleuca alternifolia*, *Leptospermum scoparium* (Myrtaceae), *Calendula officinalis* (Compositae) and *Camellia sinensis* showed that while the mouthrinse was generally well-tolerated and not associated with adverse events, it did not produce a statistically significant reduction in the abundance of oral pathogens [99].

## 12. Safety Issues Related to Phytomedicines Used in Dentistry

The clinical studies reviewed above have generally assessed the efficacy of products containing plant-derived products. However, the safety and possible side-effects of such products must also be considered. Indeed, these issues have recently been reviewed by Groppo et al. [100] in relation to natural products used in dentistry. In agreement with other studies of the clinical use of natural products, these authors have concluded that there is limited information available about the quality, safety and efficacy of herbal products used in dentistry. Given the possibility of adverse interactions between herbal formulations with conventional drugs, caution should be exercised when using herbal medicines and the need for more clinical studies is recommended. This review also considered the use of herbal products in other aspects of dentistry, including as endodontic irrigants, anti-inflammatory agents and those with sedative and anxiolytic activities.

## 13. Conclusions

As demonstrated by the examples included in this review, there is considerable evidence that plant extracts, essential oils and purified phytochemicals have the potential to be developed into agents that can be used as preventative or treatment therapies for oral diseases. While it is encouraging to see a number of clinical trials of such products, further studies of the safety and efficacy of these agents will be important to establish whether they offer therapeutic benefits, either alone or in combination with conventional therapies, that can help to reduce the overall burden of oral diseases worldwide. In particular, studies that address issues such as adequate statistical power, blinding, standardization of extracts or purified compounds, and quality control would be of great value.

## Figures and Tables

**Table 1:** Plant extracts and phytochemicals with potential application against oral bacteria.

Extract (solvent)	MIC^a^	Reference	Phytochemical (class)	MIC^a^	Reference
Propolis (ethanol)	2.0–64.0	[43]	Macrocarpals A,B,C (terpenes)	0.5–1.0	[67]
*Mikania laevigata* (ethanol)	12.5–100.0	[68]	Bakuchiol (terpene)	1.0–4.0	[57]
*Mikania glomerate* (ethanol)	12.5–100.0	[68]	Erycristagallin (flavonoid)	1.6–6.3	[48]
*Drosera peltata* (chloroform)	15.6–31.3	[25]	Beta acid	2.0	[62]
*Helichrysum italicum* (ethanol)	31.3–62.5	[40]	Xanthorrhizol (terpene)	2.0–4.0	[60]
*Coptidis rhizoma* (water)	31.0–250.0	[33]	Artocarpin (flavonoid)	3.1–12.5	[46]
*Piper cubeba* (aqueous ethanol)	90.0–200.0	[38]	Artocarpesin (flavonoid)	3.1–12.5	[46]
			Macelignan (flavonoid)	3.9	[12]
			Catechol (phenolic)	6.5	[10]
			Kuwanon G (flavonoid)	8.0	[11]
			Xanthohumol (flavonoid)	12.5	[63]
			Tetra iso-alpha acid	12.5	[62]
			Berberine (alkaloid)	13.0–20.0	[33]
			Compound 2^b^ (terpene)	15.6	[59]
					
			*Chlorhexidine* ^ c^	1.0	[49]
			*Triclosan* ^ c^	0.1–20.0	[80]

^a^Minimum inhibitory concentration (*μ*g mL^−1^); ^b^18-*β*-d-3′,6′-diacetoxyglucopyranosyl-*ent*-kaur-16-ene; ^c^The MIC values for chlorhexidine and triclosan have been added for comparative purposes.
